# Analysis of temperature adaptability of *Eocanthecona furcellata* (Wolff) (Hemiptera: Pentatomidae) based on age-stage, two-sex life table and predatory functional response

**DOI:** 10.1371/journal.pone.0344773

**Published:** 2026-03-12

**Authors:** Lingyi Liu, Mengshuang Yao, Runa Zhao, Wenlong Chen

**Affiliations:** The Provincial Key Laboratory for Agricultural Pest Management of the Mountainous Region, Scientific Observing and Experimental Station of Crop Pest in Guiyang, Ministry of Agriculture, Institute of Entomology, Guizhou University, Guiyang, Guizhou Province, China; USDA Agricultural Research Service, UNITED STATES OF AMERICA

## Abstract

*Eocanthecona furcellata* (Wolff) (Hemiptera: Pentatomidae) is a key natural predator of agricultural and forestry pests. In nature, temperature affects the growth, development and predation ability of predators. Therefore, this study assessed the growth, development, and reproduction of *E. furcellata* at 20, 23, 26, 29, and 32°C. Age-stage, two-sex life table analysis showed that the development duration of each stage decreased with increasing temperature. At 20°C, individuals reached adulthood but females did not oviposit. At 29°C, intrinsic and finite rates of increase and fecundity were higher, with values of 0.12, 1.13 and 41.59, respectively. Moreover, the mean generation time was relatively short at 29.98 d. *Spodoptera frugiperda* (Smith) (Lepidoptera: Noctuidae) is a highly destructive invasive pest that causes severe economic losses to crops. Therefore, this study evaluated the potential of *E. furcellata* to control *S. frugiperda* by predation functional response and interference effects. The functional response of adults to fourth-instar larvae of *S. frugiperda* followed the Holling II equation across all tested temperatures. Predation ability (*a/T*_*h*_) and maximum daily predation (*1/T*_*h*_) were the highest at 32°C (female:*a/T*_*h*_ = 52.7149, *1/T*_*h*_ = 51.8135; male:*a/T*_*h*_ = 46.2538, *1/T*_*h*_ = 44.8430), but adult search efficiency was negatively correlated with prey density. At constant prey density, search efficiency increased with temperature. Intraspecific competition and mutual interference were also observed among adults. Across temperatures and prey ratios, adults consistently exhibited strong predation preference for fourth-instar larvae of *S. frugiperda*. These results provide a theoretical basis for the practical use of *E. furcellata* in pest management.

## 1. Introduction

*Spodoptera frugiperda* (J.E. Smith) (Lepidoptera: Noctuidae) is a highly destructive pest characterized by broad feeding habits, high fecundity, strong adaptability, and extensive dispersal. The Food and Agriculture Organization of the United Nations categorizes it as a “globally significant agricultural pest with transboundary migratory flights” [1]. *Eocanthecona furcellata* (Wolff) (Hemiptera: Pentatomidae) is an important predatory natural enemy of agricultural and forestry pests. It preys on more than 40 pest species in the orders Lepidoptera, Coleoptera, Hymenoptera, and Hemiptera [2,3], and is now widely used to control *S. frugiperda*, *Spodoptera. litura* (Fabricius) (Lepidoptera: Noctuidae), *Plutella xylostella* L. (Lepidoptera: Plutellidae), and *Helicoverpa assulta* (Guenee) (Lepidoptera: Noctuidae), among other species [1,4]. The use of *E. furcellata* for pest control is efficient and environmentally friendly. At present, the mass rearing of *E. furcellata* incorporates provenance purification, hierarchical breeding and environmental regulation. However, owing to high breeding cost and environmental sensitivity, challenges in mass rearing and field release persist [5,6].

In nature, temperature is a major abiotic factor influencing the growth, development, and predation ability of natural enemy insects. Moreover, it strongly affects their mass propagation and field use [7–9]. Numerous studies have shown that extreme temperatures markedly affect insect growth and development [10–12]. For example, at 18°C–30°C, increasing temperature shortened the developmental duration, adult longevity, and generation cycle of *Arma chinensis* (Fallou) (Hemiptera: Pentatomidae) [13]. However, a critical knowledge gap remains regarding how temperature affects the biological traits and predatory performance of *E. furcellata*. Thus, investigating the growth, development, and predation of *E. furcellata* across temperatures to assess its developmental duration and control potential can enhance the efficiency of large-scale production during pest outbreaks and improve feasibility and field release efficiency, reduce the use of chemical pesticides, and promote green development. [14,15].

Constructing life tables is a standard and effective method for evaluating insect survival and adaptability at different temperatures [16,17]. Chi and Liu first proposed the age-stage, two-sex life table by accounting for differences between individuals and sexes [18]. Later improvements produced the user-friendly TWOSEX-MSChart, which enables accurate analysis of insect population dynami [19–21]. For example, Xu et al. used temperature as a key factor in evaluating growth and predation in *Chrysoperla sinica* (Tjeder) (Neuroptera: Chrysopidae) with the age-stage, two-sex life table, showing that spring was the best time to release the species to control *Sitobion avenae* (Fabricius) (Hemiptera:Aphididae) [9]. Similarly, Dargazani and Sahragard comprehensively evaluated the control potential of *Aphis gossypii* (Glover) (Hemiptera:Aphididae) by the introduced *Harmonia axyridis* (Pallas) (Coleoptera: Coccinellidae) in Iran using the age-stage, two-sex life table, showing that *H. axyridis* strongly suppresses *A. gossypii* [16].

When evaluating the effect of temperature on natural enemies, considering its influence on predation is essential to maximize biological control. For example, Omkar and Kumar reported that the predation rate of *Coccinella transversalis* (Fabricius) (Coleoptera: Coccinellidae) and *Coccinella septempunctata* L. (Coleoptera: Coccinellidae) increased and then declined with increasing temperature from 15°C to 35°C [22]. Fuethermore, Li et al. demonstrated that temperature modulates the instantaneous attack rate and search efficiency of natural enemies. Notably, the instantaneous attack rate of female *Scolothrips takahashii* (Thysanoptera: Thripidae) increased linearly with rising temperature [23].

The present study assessed *E. furcellata* at various temperatures: 20, 23, 26, 29, and 32°C. The age-stage, two-sex life table was used to evaluate temperature effects on the species’ growth and development. The effect of temperature on predation of *S. frugiperda* was also analyzed by fitting predation function and interference responses. Finally, the predation preference experiments were performed to explore whether the feeding of *Tenebrio molitor* L. (Coleoptera: Tenebrionidae) pupae during the feeding stage affected its predation on *S. frugiperda*. The results provide a scientific basis for indoor propagation and field release of *E. furcellata*.

## 2. Materials and methods

### 2.1 Insect rearing

Populations of *E. furcellata* were collected from maize fields at the teaching experimental field of Guizhou University, Guiyang, China (26.41°N, 106.68°E), and were subsequently reared for >10 generations in a climate chamber (58.7 cm × 51.3 cm × 185 cm, Ningbo Jiangnan Instrument Factory, Ningbo, China) with *T. molitor* pupae at 26°C ± 1°C, 70% ± 5% relative humidity (RH), and a 16:8-h light:dark photoperiod. To maintain genetic diversity, field-collected populations were regularly introduced for genetic renewal. *T. molitor* pupae were obtained from Weihai Jiulian Biotechnology Co., Ltd. (Shandong, China) and reared on a wheat bran diet. Larvae were maintained at 26°C and 55%–60% RH, fed wheat bran, and reared for 5–6 weeks before pupae were separated from bran and frass. *S. frugiperda* larvae was collected in June 2023 from a maize field in Longping, Luodian County, Guizhou Province, China (25.48°N, 106.63°E).

### 2.2 Test methods

Experiments were conducted at five constant temperatures (20, 23, 26, 29, or 32°C) and 70% ± 5% RH under a 16:8-h light:dark photoperiod. At each temperature, *E. furcellata* was reared in a climate chamber from eggs to adults for subsequent tests.

#### 2.2.1 Effects of temperature on growth, development, and reproduction in *E. furcellata.*

Groups of 100 eggs (successfully tracked) were placed in climate chambers at each temperature. After the group of eggs hatched, first-instar nymphs were individually placed in feeding boxes (6.5 cm in diameter and 3.5 cm in height) and reared at each temperature. Each instar was marked with a serial number and provided with a wet cotton ball and *T. molitor* pupae (first instars were given only wet cotton balls, as they do not prey). Sex was noted, and molting, mortality, and eclosion were observed and recorded for each *E. furcellata* individual daily, with food replenished as needed and feeding boxes cleaned and replaced. After adult emergence, one newly emerged female and one male were paired in a clean feeding box (15.2-cm length, 9.9-cm width, and 6.0-cm height; consistent in the experiments described below) and supplied daily with wet cotton balls and fresh *T. molitor* pupae. Oviposition date, egg count, and time of death were recorded.

#### 2.2.2 Predatory function and search efficiency of *E. furcellata* on *S. frugiperda* at different temperatures.

Based on pre-experiments, density gradients of fourth-instar *S. frugiperda* larvae were set at 5, 10, 15, and 20 larvae per box. A wet cotton ball and fresh maize leaves were added to each box to reduce *S. frugiperda* larval cannibalism. One 24 h-starved *E. furcellata* reared at the corresponding temperature was introduced into each box, which was then placed in a climate chamber at that temperature. Male and female adults were tested separately. Each prey density treatment was repeated five times. After 24 h, the number of surviving prey larvae in each box was recorded.

#### 2.2.3 Effect of *E. furcellata* density on predation of *S. frugiperda* at different temperatures.

Based on pre-experiments, *E. furcellata* adult densities were set at 1, 2, 3, 4, and 5 individuals per box, and each individual was starved for 24 hours before the experiment. Both sexes reared at the target temperatures were used, with males and females tested separately. Each box received 60 fourth-instar *S. frugiperda* larvae, a wet cotton ball, and sufficient maize leaves to reduce larval self-injury. Boxes were then placed in climate chambers at the corresponding temperatures. Each treatment density was repeated five times. After 24 h, the number of surviving larvae in each box was recorded.

#### 2.2.4 Predation preference of *E. furcellata* between *S. frugiperda* and *T. molitor* at different temperatures.

Based on pre-experiments, three prey ratios were tested: *S. frugiperda*:*T. molitor* at 10:20, 15:15, and 20:10 individuals per box. Male and female adults were tested separately. Different ratios of fourth-instar *S. frugiperda* larvae and *T. molitor* pupae were placed in feeding boxes, and one 24 h-starved *E. furcellata* adult reared at each temperature was introduced. Prey were counted after 24 h. Each treatment was repeated five times. All experiments were conducted in artificial climate chambers.

### 2.3 Data analysis

Parameters of the age-stage, two-sex life table were estimated using the bootstrap method in TWOSEX-MSChart (Chi and Liu 1985) with 100,000 resampled data values [17]. Development time, survival rate, and female daily fecundity were analyzed using this software [24,25]. The same statistical methods were applied to test differences in the age-stage, two-sex life table-related parameters. GraphPad Prism 10 was used to produce histograms and line charts, SigmaPlot 15 was used to create *s*_*xj*_, *l*_*x*_, *m*_*x*_, *l*_*x*_*m*_*x*_, *e*_*xj*_, and *v*_*xj*_ curves (defined below), and Excel 2021 was used to record and process raw predation-related data. A chi-square test was applied to further validate the predation of *E. furcellata* against *S. frugiperda*. Degrees of freedom (*df*) were calculated as $df=N_{g}-N_{p}-\text{1}\;$ (where *N*_*g*_ represents the number of groups and *N*_*p*_ is the number of model parameters), and significance was defined as *P* < 0.05. When *χ²* < *χ²*_0.05, *df*_ and *P* > 0.05, the model was considered well fitted, with no significant difference between theoretical values and observed values. The functional response was fitted using the Holling II disk equation. Predation parameters were estimated via the least-squares method, 95% confidence intervals were calculated, and predation charts were created using GraphPad Prism 10.

#### 2.3.1 Formulae for life table parameters.

(1) The age-specific survival rate (*l*_*x*_), i.e., survival from egg to age *x*, where *S*_*xj*_ is the age-stage-specific survival rate (probability of *E. furcellata* surviving from egg to age *x* stage *j*), was calculated as follows

$$l_{x\;}=\;\sum_{j\;=\;\text{1}}^{m}s_{xj}.$$

(2) The age-specific fecundity rate (*m*_*x*_), i.e., average fecundity of the *E. furcellata* population at age *x*, where *f*_*xj*_ is age-specific fecundity in female adults (eggs laid at age *x* stage *j*), was determined as follows:

$$m_{x}\;=\;\sum_{j\;=\;\text{1}}^{m}s_{xj}f_{xj}/\sum_{j\;=\;\text{1}}^{m}s_{xj}.$$

(3) The population age-specific reproduction value (*l*_*x*_*m*_*x*_), i.e., product of *l*_*x*_ and *m*_*x*_, was calculated as follows:

$$l_{x}m_{x}\;=\;\sum_{j\;=\;\text{1}}^{m}s_{xj}f_{xj}.$$

(4) The age-stage life expectancy (*e*_*xj*_), i.e., expected days of survival for *E. furcellata* at age *x* stage *y*, where *n* is the last population age for each treatment, and *s*’_*iy*_ is the probability that an individual of age *x* stage *y* survives to age *i* stage *j*, was determined using the following formula:

$$e_{xj}\;=\;\sum_{i\;=\;x}^{n}\sum_{j\;=\;y}^{m}s_{iy}^{\prime }.$$

(5) The age-stage reproductive capacity (*v*_*xj*_), i.e., contribution of an *E. furcellata* individual at age *x* stage *y* to the population’s future, was calculated as follows:

$$v_{xj}\;=\;\frac{e^{r(x\;+\;\text{1})}}{s_{xj}}\sum_{i\;=\;x}^{n}e^{-r(i\;+\;\text{1})}\sum_{j\;=\;y}^{m}s_{iy}^{\prime }f_{iy}.$$

(6) The intrinsic rate of increase (*r*), i.e., the maximum instantaneous population growth rate under stable conditions (specific physical and biological conditions), was determined as follows:

$$\sum_{x\;=\;\text{0}}^{\infty }e^{-r(x\;+\;\text{1})}l_{x}m_{x}\;=\;\text{1}.$$

(7) The finite rate of increase (*λ*), i.e., average daily growth rate of the *E. furcellata* population without external environmental limitations, was calculated as follows:

$$\lambda \;=\;e^{r}.$$

(8) The net reproductive rate (*R*_*0*_), i.e., total offspring produced per individual in a lifetime, was determined using the following formula:

$$R_{\text{0}}\;=\;\sum_{x\;=\;\text{0}}^{\infty }l_{x}m_{x}.$$

(9) The average generation period (*T*), i.e., time required for successive generations under stable growth, was calculated as follows:

$$T\;=\;\frac{\text{ln}R_{\text{0}}}{r}.$$

(10) The gross reproduction rate, i.e., the sum of *m*_*x*_ values, was determined as follows:

$$GRR\;=\;\sum m_{x}.$$

#### 2.3.2 Formula for predation experiment.

The predator–prey functional response was modeled using the Holling II type disk equation:


$$N_{A}\;=\;aNT_{r}/\left(\text{1}\;+\;aT_{h}N\right),$$

where *N*_*a*_ represents the number of fourth-instar *S. frugiperda* larvae preyed upon, *a* is the instantaneous attack rate of *E. furcellata* toward *S. frugiperda*, *N* is the set prey density, *T*_*r*_ is the total duration, and *T*_*h*_ represents the handling time of *E. furcellata* per to *S. frugiperda* larva [26,27].

The search effect formula is as follows:


S = aT/(1 + aThN),

where *S* is the search effect value, and other parameters are defined above [28].

The interference effect formula is as follows:


$$E\;=\;QP^{-m},E\;=\;\frac{N_{a}}{NP}.$$

The Hassell–Verley model was used to fit self-density interference. In the above formula, *E* is the predation rate, *N*_*a*_ the number of prey consumed, *N* the initial prey count, *P* the initial predator density, *Q* the search coefficient, and *m* the interference coefficient [29].

The selectivity index (*D*) of *E. furcellata* toward prey was determined as follows:


$$D\;=\;N_{P\text{1}}N_{\text{2}}/N_{P\text{2}}N_{\text{1},}$$

where *N*_*1*_ and *N*_*2*_ are initial *S. frugiperda* and *T. molitor* densities, respectively, and *N*_*p1*_ and *N*_*p2*_ are the *S. frugiperda* and *T. molitor* prey consumed, respectively. When *D* > 1, predators showed preference for a given prey [30].

## 3. Results

### 3.1 Effects of temperature on growth, development, and reproduction in *E. furcellata*

Between 20°C and 32°C, developmental duration and preadult stages of *E. furcellata* shortened with increasing temperature. Male and female adults lived significantly longer at 20°C compared with other temperatures. Both the adult preoviposition period and total preoviposition period were shortest at 29°C. Average fecundity increased with temperature, peaking at 32°C but differences from 26°C and 29°C were not significant (Table 1).

**Table 1:** Development time, adult longevity, total longevity, APOP, TPOP, and fecundity of *E. furcellata* at different temperatures.

Stage	Temperature(°C)				
	20	23	26	29	32
Egg (d)	14.00 ± 0.00a	11.81 ± 0.04b	6.69 ± 0.07c	4.84 ± 0.08d	4.17 ± 0.08e
First instar nymphs (d)	6.04 ± 0.03a	3.91 ± 0.05b	3.26 ± 0.06c	2.52 ± 0.07d	2.12 ± 0.08e
Second instar nymphs (d)	9.05 ± 0.26a	7.36 ± 0.26b	4.13 ± 0.09c	3.66 ± 0.08d	2.99 ± 0.11e
Third instar nymphs (d)	7.63 ± 0.37a	4.78 ± 0.16b	3.41 ± 0.10c	2.00 ± 0.07d	2.02 ± 0.11d
Fourth instar nymphs (d)	10.56 ± 0.75a	4.92 ± 0.13b	4.26 ± 0.13c	2.60 ± 0.07e	2.95 ± 0.13d
Fifth instar nymphs (d)	15.20 ± 1.56a	7.95 ± 0.28b	6.20 ± 0.26c	4.05 ± 0.13d	4.20 ± 0.26d
Preadult Duration(d)	61.60 ± 3.20a	40.62 ± 0.45b	27.55 ± 0.34c	19.66 ± 0.19d	18.34 ± 0.30e
Adult (d)	17.20 ± 2.63c	33.59 ± 3.04a	22.35 ± 2.09b	16.99 ± 1.05c	16.09 ± 1.34c
Total longevity (female, d)	78.75 ± 3.12a	72.95 ± 4.56b	50.86 ± 3.79c	37.25 ± 1.64d	36.63 ± 1.71d
Total longevity (male, d)	79.00 ± 3.25a	75.71 ± 4.24b	49.11 ± 2.36c	36.14 ± 1.44d	32.38 ± 1.85d
APOP (d)	–	18.29 ± 1.59a	9.50 ± 0.87b	6.56 ± 0.67b	8.29 ± 0.39b
TPOP (d)	–	59.53 ± 8.90a	37.79 ± 1.15b	26.04 ± 0.88c	26.95 ± 0.59c
Fecundity (total eggs/female)	–	98.24 ± 15.04b	152.21 ± 18.34a	166.36 ± 21.20a	168.38 ± 22.75a
Oviposition days (d)	–	2.47 ± 0.40b	4.29 ± 0.70a	3.80 ± 0.44a	4.19 ± 0.52a

Note: Data in the table represent mean ±SE. Different letters within the same row indicate significant differences among different temperatures (paired bootstrap test, *P* < 0.05). Standard errors were estimated through 100,000 bootstrap resampling. APOP, adult preoviposition period; TPOP, total preoviposition period.

At 29°C, *R*_*0*_ reached 41.59, *r* was 0.12, and *λ* was 1.13, which are higher than those at other temperatures and significantly higher than at 23°C. *T* was significantly longer at 20°C compared with the other temperatures. *GRR* peaked at 26°C but did not differ significantly across treatments (Table 2).

**Table 2:** Life table parameters of *E. furcellata* at different temperatures.

	Temperature(°C)				
	20	23	26	29	32
Net reproductive rate *R*_*0*_	–	16.70 ± 4.44b	21.31 ± 5.83ab	41.59 ± 8.90a	35.36 ± 8.33a
Mean generation time *T* (d)	89.00 ± 3.29a	65.12 ± 2.38b	43.65 ± 2.21c	29.98 ± 0.99d	31.38 ± 0.51d
Intrinsic rate of increase *r* (d^-1^)	–	0.03 ± 0.01c	0.07 ± 0.01b	0.12 ± 0.01a	0.11 ± 0.01a
Finite rate of increase *λ* (d^-1^)	1.00 ± 0.01d	1.04 ± 0.01c	1.07 ± 0.01b	1.13 ± 0.01a	1.12 ± 0.01a
Gross reproductive rate	–	126.24 ± 33.57a	138.75 ± 38.30a	136.39 ± 48.77a	134.92 ± 26.10a

Note: Data in the table represent mean ±SE. Different letters within the same row indicate significant differences among different temperatures (paired bootstrap test, *P* < 0.05). Standard errors were estimated through 100,000 bootstrap resampling.

**Fig 1:**
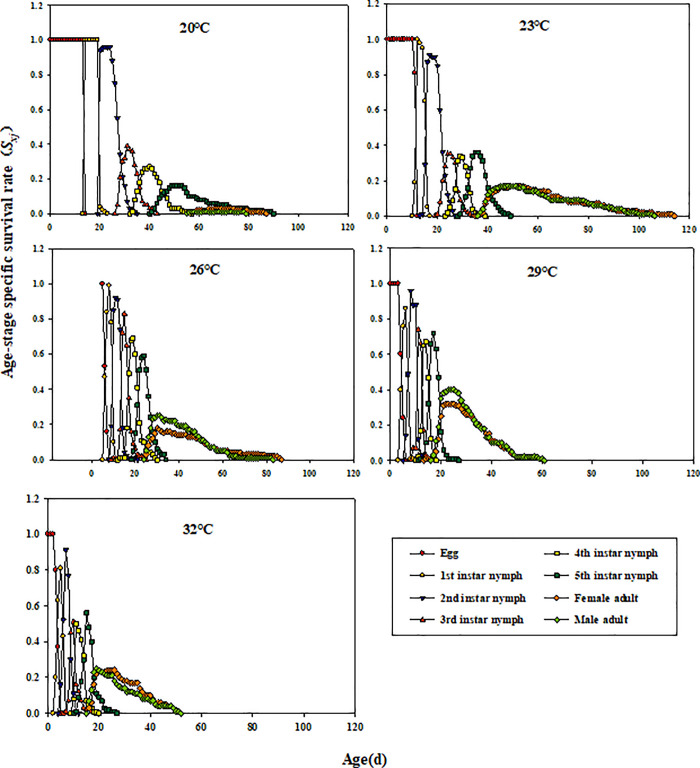
Effects of different temperatures on the age-stage-specific survival rate (*s*_*xj*_) of *E. furcellata.*

Stage overlap was pronounced in *E. furcellata*. Except for eggs, survival rates increased and then declined across instars. At 23°C, adults survived longest, with males and females dying at 115 and 106 days, respectively (Fig 1).

**Fig 2:**
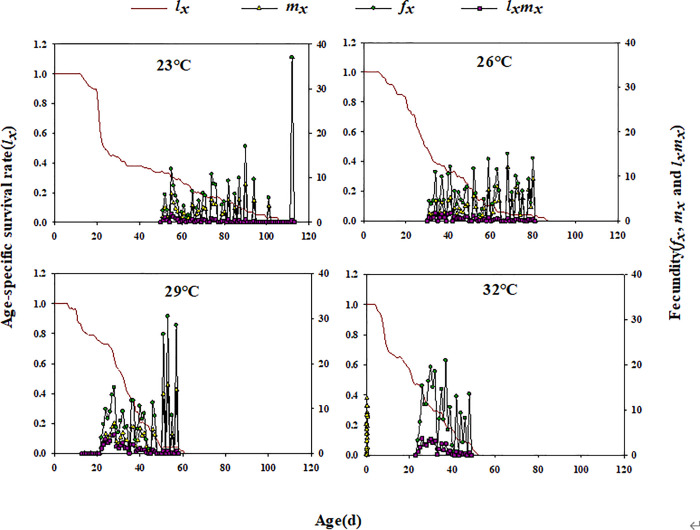
Effects of different temperatures on the age-specific survival rate (*l*_*x*_), age-stage-specific fecundity (*f*_*x*_), age-specific fecundity (*m*_*x*_), and age-specific maternity (*l*_*x*_*m*_*x*_) of *E. furcellata.*

**Fig 3:**
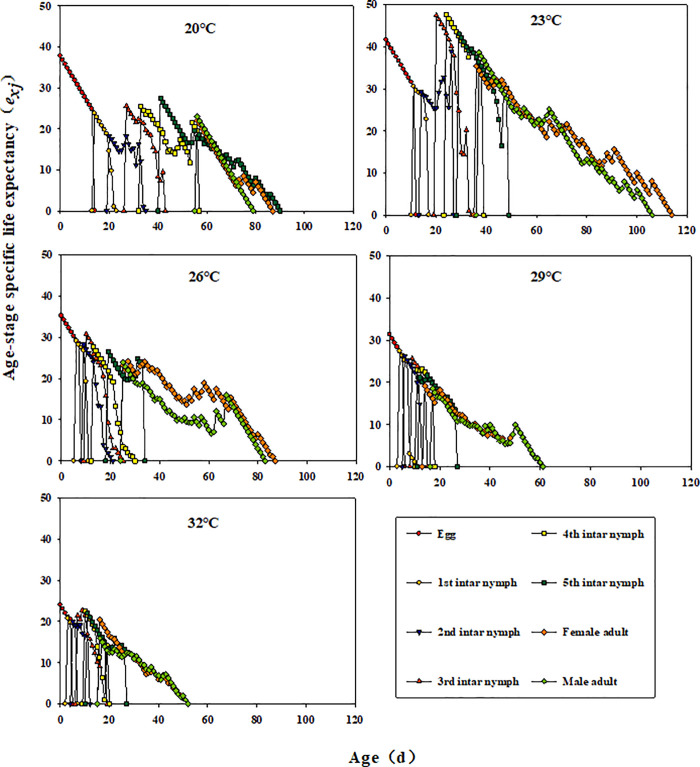
Effects of different temperatures on the age-stage-specific life expectancy (*e*_*xj*_) of *E. furcellata.*

**Fig 4:**
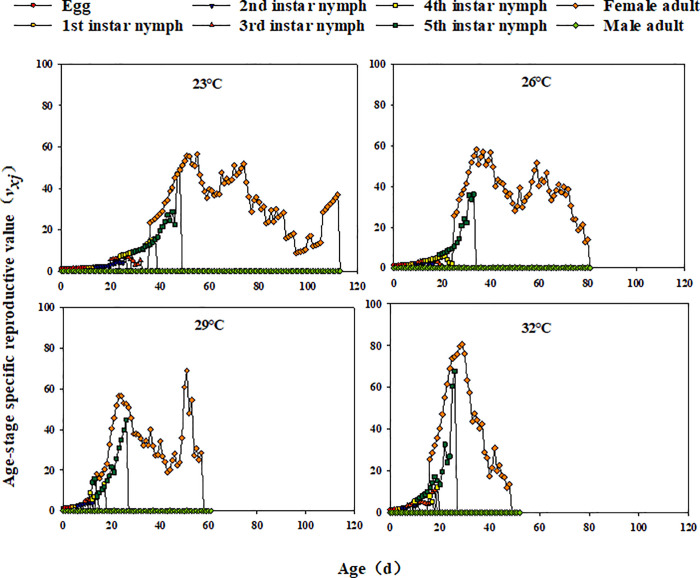
Effects of different temperatures on the age-stage-specific reproductive values (*v*_*xj*_) of *E. furcellata.*

Across temperatures, *l*_*x*_ declined with *E. furcellata* age. Additionally, *f*_*x*_ and *m*_*x*_ fluctuated, showing a pattern of increase, decline, and eventual decrease to 0 (Fig 2). Except for eggs and first-instar nymphs at all temperatures and second to fourth instars at 26°C, *e*_*xj*_ showed a general trend of decreasing, increasing, and then declining with development time (Fig 3). Similarly, *v*_*xj*_ in *E. furcellata* increased with developmental duration, with peak values increasing at higher temperatures (Fig 4).

### 3.2 Predatory function and search efficiency of *E. furcellata* on *S. frugiperda* at different temperatures

**Fig 5:**
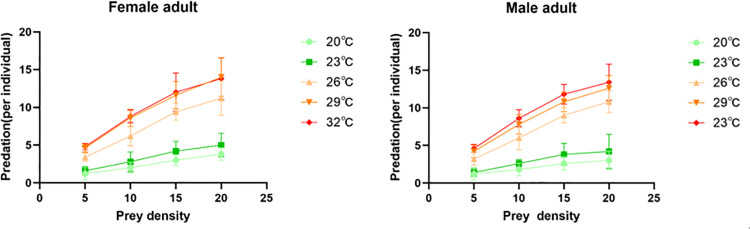
Predatory functional response of *E. furcellata* adults on the fourth instar larvae of *S. frugiperda* at different temperatures.

Predation by both *E. furcellata* sexes on *S. frugiperda* generally increased with prey density (Fig 5). Chi-square tests (*x*^2^ = 1.5, *df* = 17) are less than critical values (*x*^2^_0.05, 17_ = 27.59), then *P* > 0.05, suggesting no significant difference between theoretical and actual values. Affirming the functional response to fourth-instar *S. frugiperda* larvae fitted the Holling II model well (Table 3), indicating that *E. furcellata* predation was significantly correlated with prey density across temperatures. For female *E. furcellata*, the highest instantaneous attack rate on *S. frugiperda* (*a* = 1.0223) occurred at 29°C. Predation ability (*a/T*_*h*_) and maximum daily consumption (*1/T*_*h*_) increased with temperature, peaking at 32°C (52.7149 and 51.8135, respectively). Male predation parameters followed the same trend, with maxima at 32°C (*a* = 1.0315, *a/T*_*h*_ = 46.2538, and *1/T*_*h*_ = 44.8430). Handling time (*T*_*h*_) decreased as temperature increased. Thus, temperature had a significant effect on *E. furcellata* predation in *S. frugiperda*: predation efficiency was low at 20°C–23°C and high at 26°C–32°C.

**Table 3:** The functional response models and parameters of *E. furcellata* adult on the fourth instar larvae of *S. frugiperda* at different temperature.

	Temperature (°C)	Functional response equation	*R*^*2*^ Correlation efficient	*a* Instantaneous attack rate	*a* 95% Confidence intervals	*T*_h_ Handing time (d)	*T*_h_ 95% Confidence intervals	*a/T*_h_ Predation capacity	*1/T*_h_ Daily maximum predation number
Female	20	*N*_a_ = 0.2803*N*/(1 + 0.0357*N*)	0.9916	0.2803	(0.205, 0.420)	0.1273	(0.088, 0.190)	2.2020	7.8555
	23	*N*_a_ = 0.3567*N*/(1 + 0.0247*N*)	0.9894	0.3567	(0.285, 0.480)	0.0693	(0.055, 0.095)	5.1477	14.4300
	26	*N*_a_ = 0.7475*N*/(1 + 0.0207*N*)	0.9907	0.7475	(0.620, 0.920)	0.0277	(0.023, 0.034)	26.9854	36.1011
	29	*N*_a_ = 1.10223*N*/(1 + 0.0214*N*)	0.9993	1.0223	(0.950, 1.120)	0.0209	(0.019, 0.023)	48.9132	47.8469
	32	*N*_a_ = 1.0174*N*/(1 + 0.0196*N*)	0.9972	1.0174	(0.920, 1.150)	0.0193	(0.017, 0.022)	52.7149	51.8135
Male	20	*N*_a_ = 0.3005*N*/(1 + 0.0541*N*)	0.9839	0.3005	(0.235, 0.410)	0.1800	(0.135, 0.250)	1.6693	5.5556
	23	*N*_a_ = 0.3072*N*/(1 + 0.0187*N*)	0.9970	0.3072	(0.280, 0.335)	0.0609	(0.055, 0.068)	5.0439	16.4204
	26	*N*_a_ = 0.7116*N*/(1 + 0.0223*N*)	0.9889	0.7116	(0.605, 0.850)	0.0313	(0.027, 0.037)	22.7346	31.9489
	29	*N*_a_ = 0.9331*N*/(1 + 0.0214*N*)	0.9990	0.9331	(0.870, 1.000)	0.0229	(0.021, 0.025)	40.7466	43.6681
	32	*N*_a_ = 1.0315*N*/(1 + 0.0230*N*)	0.9977	1.0315	(0.950, 1.130)	0.0223	(0.020, 0.025)	46.2538	44.8430

Note: *N*_*a*_ represents the number of prey for *E. furcellata*, *N* represents the density of *S. frugiperda*.

**Fig 6:**
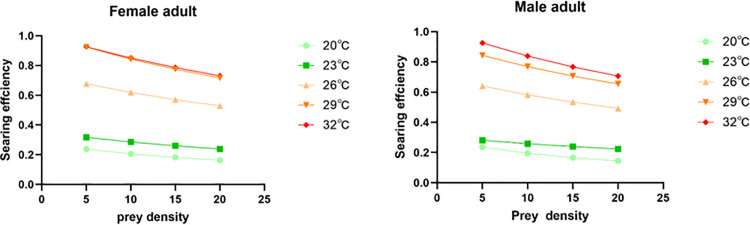
Searching efficiency of *E. furcellata* adults on the fourth instar larvae of *S. frugiperda* at different temperatures.

Search efficiency in *E. furcellata* was negatively correlated with *S. frugiperda* prey density at all temperatures. At a constant temperature, *E. furcellata* searching decreased with increasing prey density; at a constant prey density, search efficiency increased with increasing temperature. These results indicate that temperature had a significant effect on the search efficiency of *E. furcellata* preying on *S. frugiperda* (Fig 6).

### 3.3 Effect of *E. furcellata* density on predation of *S. frugiperda* at different temperatures

In a defined space, increasing predator density often induces considerable intraspecific interactions, a phenomenon termed mutual interference. This interference reduces the foraging efficiency of individual predators as density increases. The Hassell–Verley interference model was applied to fit the predation rate of *E. furcellata* adults on fourth-instar *S. frugiperda* larvae under varying temperatures and predator densities. The correlation coefficient between predation rate and predator density was *R*^*2*^ > 0.97, and chi-square tests values were below the 0.05 critical threshold (*χ²* < *χ²*_0.05, 17_), indicating *P* > 0.05 and strong agreement between observed and theoretical values. The model effectively characterized the mutual interference of *E. furcellata* during predation.At constant predator density, *E. furcellata* predation on fourth-instar *S. frugiperda* larvae increased with temperature. Higher predator densities increased the total prey killed and scramble competition intensity but reduced the predation rate and per-capita predation, indicating competition and mutual interference among individuals with a specific spatial range. (Tables 4 and 5).

**Table 4:** Predation rate of *E. furcellata* adults on fourth instar larvae of *S. frugiperda.*

The density of *E. furcellata*												
	Temperature(°C)	1		2		3		4		5		*χ²*
		Na	E	Na	E	Na	E	Na	E	Na	E	
Female	20	8.00	0.13	7.60	0.06	6.27	0.03	5.40	0.02	4.76	0.02	1.20
	23	11.60	0.19	9.20	0.08	7.20	0.04	6.45	0.03	5.48	0.02	8.47
	26	15.20	0.25	13.40	0.11	10.60	0.06	8.90	0.04	7.56	0.03	13.00
	29	21.20	0.35	20.10	0.17	16.07	0.09	12.55	0.05	10.68	0.04	22.43
	32	23.80	0.40	22.90	0.19	17.47	0.10	12.95	0.05	10.76	0.04	26.86
Male	20	6.60	0.11	6.50	0.05	5.87	0.03	4.50	0.02	4.12	0.01	2.54
	23	9.00	0.15	8.60	0.07	6.87	0.04	5.55	0.02	5.12	0.02	4.94
	26	12.20	0.20	11.90	0.10	9.93	0.06	8.30	0.03	7.72	0.03	5.26
	29	17.40	0.29	17.30	0.14	12.13	0.07	9.70	0.04	8.52	0.03	21.67
	32	20.40	0.34	18.20	0.15	13.07	0.07	10.20	0.04	9.00	0.03	15.89

Note: *E* is the predation rate, *N*_*a*_ the number of predation.

**Table 5:** Equations and estimated parameters of interferential effect to *E. furcellata* adults.

	Temperature(°C)	*Q* Searching coefficient	*m* Disturbance coefficient	Hassell-Verley model	*R* ^*2*^
Female	20	0.1432	0.3270	*E* = 0.1432*P*^-0.3270^	0.9923
	23	0.1999	0.4620	*E* = 0.1999*P*^-0.4620^	0.9982
	26	0.2723	0.4350	*E* = 0.2723*P*^-0.4350^	0.9932
	29	0.3925	0.4310	*E* = 0.3925*P*^-0.4310^	0.9853
	32	0.4523	0.5030	*E* = 0.4523*P*^-0.5030^	0.9793
Male	20	0.1203	0.2990	*E* = 0.1203*P*^-0.2990^	0.9845
	23	0.1631	0.3720	*E* = 0.1631*P*^-0.3720^	0.9897
	26	0.2188	0.3001	*E* = 0.2188*P*^-0.3001^	0.9913
	29	0.3272	0.4730	*E* = 0.3272*P*^-0.4730^	0.9820
	32	0.3748	0.5350	*E* = 0.3748*P*^-0.5350^	0.9890

### 3.4 Predation preference of *E. furcellata* between *S. frugiperda* and *T. molitor* at different temperatures

The predation preference of *E. furcellata* for fourth-instar *S. frugiperda* larvae and *T. molitor* pupae at different temperatures is presented in Tables 6 and 7. When *S. frugiperda* and *T. molitor* coexisted in varying proportions, the selectivity index of male and female *E. furcellata* adults for fourth-instar *S. frugiperda* larvae was > 1 in all three combinations. This indicates that both *E. furcellata* sexes exhibited a strong predation preference for fourth-instar *S. frugiperda* larvae.

**Table 6:** Predation preference of female *E. furcellata* adults on fourth instar larvae of *S. frugiperda* and *T.molitor* pupae.

Temperature(°C)	Prey number (individual)	Predation number		*D* Prey selectivity index
		*S. frugiperda*	*T.molitor*	
20	10:20	2.20 ± 1.327	1.40 ± 0.490	3.143
	15:15	3.20 ± 0.980	1.00 ± 0.633	3.200
	20:10	4.20 ± 1.166	0.80 ± 0.400	2.625
23	10:20	4.20 ± 1.327	2.20 ± 1.166	3.818
	15:15	4.60 ± 1.020	2.00 ± 0.894	2.300
	20:10	5.60 ± 1.020	1.60 ± 0.490	1.750
26	10:20	5.00 ± 1.095	2.80 ± 1.166	3.571
	15:15	5.40 ± 1.200	2.60 ± 0.800	2.077
	20:10	6.40 ± 1.020	2.20 ± 0.748	1.455
29	10:20	7.00 ± 1.789	3.80 ± 1.600	3.684
	15:15	7.60 ± 1.357	3.60 ± 1.020	2.111
	20:10	8.20 ± 1.327	3.20 ± 0.748	1.281
32	10:20	7.80 ± 1.166	4.20 ± 0.748	3.714
	15:15	8.20 ± 1.470	3.80 ± 0.748	2.158
	20:10	8.80 ± 1.166	3.20 ± 0.748	1.375

Note: 10:20 represent the prey ratios of fourth instar larvae of *S. frugiperda*: *T. molitor* pupae = 10:20. 15:15 represent the prey ratios of fourth instar larvae of *S. frugiperda*: *T. molitor* pupae = 15:15. 20:10 represent the prey ratios of fourth instar larvae of *S. frugiperda*: *T. molitor* pupae = 20:10. Data are presented as means ± standard errors of five replicates.

**Table 7:** Predation preference of male *E. furcellata* adults on fourth instar larvae of *S. frugiperda* and *T.molitor* pupae.

Temperature(°C)	Prey number (individual)	Predation number		*D* Prey selectivity index
		*S. frugiperda*	*T.molitor*	
20	10:20	2.20 ± 0.748	1.40 ± 0.800	3.143
	15:15	3.40 ± 1.357	1.20 ± 0.400	2.833
	20:10	3.80 ± 1.470	0.60 ± 0.489	3.167
23	10:20	4.00 ± 1.095	2.00 ± 0.894	4.000
	15:15	4.20 ± 1.166	2.00 ± 0.633	2.100
	20:10	5.20 ± 1.939	1.40 ± 0.490	1.857
26	10:20	4.40 ± 1.020	2.20 ± 0.748	4.000
	15:15	4.60 ± 1.020	2.20 ± 0.980	2.909
	20:10	6.00 ± 1.789	1.80 ± 0.400	1.667
29	10:20	6.40 ± 1.357	4.00 ± 1.414	3.200
	15:15	7.40 ± 1.497	3.20 ± 1.327	2.313
	20:10	8.00 ± 1.673	2.60 ± 1.356	1.539
32	10:20	6.60 ± 1.357	4.20 ± 0.748	3.143
	15:15	7.80 ± 1.327	3.60 ± 1.019	2.167
	20:10	8.80 ± 1.166	3.00 ± 0.633	1.467

Note: 10:20 represent the prey ratios of fourth instar larvae of *S. frugiperda*: *T. molitor* pupae = 10:20. 15:15 represent the prey ratios of fourth instar larvae of *S. frugiperda*: *T. molitor* pupae = 15:15. 20:10 represent the prey ratios of fourth instar larvae of *S. frugiperda*: *T. molitor* pupae = 20:10. Data are presented as means ± standard errors of five replicates

## 4. Discussion

Studying the effects of temperature on the growth, development, and reproduction of natural enemy insects is critical for ecological regulation of field pests and indoor artificial mass-rearing, particularly for improving biological control efficiency [31]. In the present study, at 20°C, eggs hatched and developed into adults but female adults failed to oviposit, consistent with the findings of Zhu [32]. This may be due to prolonged preoviposition periods and shortened female lifespan at 20°C, causing death before egg laying. In addition, Xu et al. reported that egg and nymph development in *A. chinensis* was significantly shortened as temperature increased from 18°C to 30°C [33]. Similarly, the development of each instar of *Picromerus bidens* L. and *Picromerus lewisi* (Fallou) decreased as temperature increased from 18°C to 32°C, and *P. bidens* eggs failed to hatch normally at 15°C and 35°C [34,35].

Population parameters are key for evaluating treatment effects on insects [36]. The current study found that *E. furcellata* completed development at 23°C with long survival but low predation, indicating that eggs can be stored at this temperature during winter conservation. Prolonging *E. furcellata* development at 23°C extends conservation periods and reduces feeding costs. Thus, relatively low temperatures in practical applications can prolong *E. furcellata* shelf life and reduce mortality due to untimely feeding during transport. At 26°C, the developmental duration of each instar was moderate, population fecundity was high, and adult longevity was prolonged, with *E. furcellata* completing 8–9 generations annually. Compared with 26°C, *r*, *λ*, and fecundity were higher at 29°C, with a shorter generation cycle, indicating that this temperature is more favorable for large-scale propagation of *E. furcellata*.

For *E. furcellata*, predation under five constant temperatures followed the Holling II model, consistent with previous studies on natural enemy insects on young *S. frugiperda* larvae [37–45]. Predation of *E. furcellata* adults toward *S. frugiperda* larvae increased with temperature. When the temperature was 32°C, the *a/Th* and *1/Th* of female adults were the highest, which were 52.7149 and 51.8135, respectively. The *a/Th* and day *1/Th* of male adults were also the highest, 46.2538 and 44.8430, respectively,

consistent with studies assessing 17°C–37°C treatments [46]. These adults showed the strongest predation at high temperatures, whereas low temperatures limited activity and control efficiency.

The search efficiency of *E. furcellata* adults negatively correlated with *S. frugiperda* prey density at each temperature. Additionally, the prey search efficiency of *E. furcellata* adults increased with temperature, being lowest at 20°C. At temperatures above 29°C and high prey densities, search efficiency almost plateaued or began to decline, likely due to thermal inhibition of *E. furcellata* predatory activity, similar to *Neoseiulus californicus* (McGregor) preying on *Eutetranychus chusorientalis* (Klein), wherein efficiency decreased above 33°C [47].

Intraspecific competition and mutual interference occurred among adult *E. furcellata* at the same prey density, increasing with predator density. Search as well as mutual interference coefficients increased with temperature, demonstrating that predation is closely linked to predator density, consistent with the findings of Fan et al. and Tang et al. [1,39]. In the current study, when both fourth-instar *S. frugiperda* larvae and *T. molitor* pupae were present, adult *E. furcellata* consistently preferred *S. frugiperda*, indicating that feeding *T. molitor* in the laboratory will not reduce its predation and control of *S. frugiperda*.

## 5. Conclusions

In summary, the storage of *E. furcellata* can effectively prolong its shelf life while reducing rearing costs. Future storage experiments for this species could thus be conducted at approximately 23°C, whereas 29°C favors large-scale propagation. In this study, *S. frugiperda* was selected as prey owing to its wide distribution, adaptability, rapid reproduction, and severe effects on crops and forests [4]. The current findings support biological pest control concepts, showing that *E. furcellata* adults effectively prey on fourth-instar *S. frugiperda* larvae, with the control being strongest at 32°C and weakest at 20°C. Therefore, we speculate that low-temperature releases of *E. furcellata* require caution, whereas high-temperature summer outbreaks of *S. frugiperda* can be managed using *E. furcellata* in the field, the specific results need to be verified by subsequent field experiments. Optimal control should consider actual prey density and appropriate predator release rates. Predation preference experiments confirmed strong adult *E. furcellata* preference for *S. frugiperda*, providing guidance for mass propagation and field biological control. Because this study was conducted under constant indoor temperatures, future investigations should incorporate field experiments.

## Supporting information

S1 File Supporting information. (ZIP)
